# Simulation of Intra-Aneurysmal Blood Flow by Different Numerical Methods

**DOI:** 10.1155/2013/527654

**Published:** 2013-04-15

**Authors:** Frank Weichert, Lars Walczak, Denis Fisseler, Tobias Opfermann, Mudassar Razzaq, Raphael Münster, Stefan Turek, Iris Grunwald, Christian Roth, Christian Veith, Mathias Wagner

**Affiliations:** ^1^Department of Computer Science VII, Dortmund University of Technology, Dortmund, Germany; ^2^Institute of Applied Mathematics, LS III, Dortmund University of Technology, Dortmund, Germany; ^3^Oxford Biomedical Research Centre, University of Oxford, Oxford, UK; ^4^Department of Neuroradiology, University of Saarland Medical School, Homburg Saar, Germany; ^5^Department of Pathology, University of Saarland Medical School, Homburg Saar, Germany

## Abstract

The occlusional performance of sole endoluminal stenting of intracranial aneurysms is controversially discussed in the literature. Simulation of blood flow has been studied to shed light on possible causal attributions. The outcome, however, largely depends on the numerical method and various free parameters. The present study is therefore conducted to find ways to define parameters and efficiently explore the huge parameter space with finite element methods (FEMs) and lattice Boltzmann methods (LBMs). The goal is to identify both the impact of different parameters on the results of computational fluid dynamics (CFD) and their advantages and disadvantages. CFD is applied to assess flow and aneurysmal vorticity in 2D and 3D models. To assess and compare initial simulation results, simplified 2D and 3D models based on key features of real geometries and medical expert knowledge were used. A result obtained from this analysis indicates that a combined use of the different numerical methods, LBM for fast exploration and FEM for a more in-depth look, may result in a better understanding of blood flow and may also lead to more accurate information about factors that influence conditions for stenting of intracranial aneurysms.

## 1. Introduction

 The accurate incidence and prevalence of unruptured nonaortic aneurysms of 3 mm or less in diameter is controversially discussed. The likelihood of detection is increasing with improved imaging techniques [1, 2]. Among the risk factors are age, hypertension, and the habit of cigarette smoking [3]. Size and perhaps geometry of the aneurysm contribute to the risk of rupture which may be less than 5% per year [4]. A rupture of an intracranial aneurysm can cause devastating subarachnoid hemorrhage with high morbidity and mortality [5]. For the treatment of unruptured aneurysms, there is a selection of endovascular and surgery-based treatment modalities, for which the risks and rates of complication have been described elsewhere [3]. Hemorrhage as a consequence of ruptured intracranial aneurysms can be prevented by means of minimally invasive therapy, endoluminal stenting.

In the last few years, endovascular treatment of intracranial aneurysms has become a possible minimal invasive alternative to neurosurgical therapy which was until then unequalled. The aneurysm is treated with electrolytically detachable coils, the use of which is limited for wide-necked aneurysms. It is often impossible to coil an aneurysm after stent placement, so the treatment of the aneurysm with a covered or small-cell-designed stent that would permit an immediate occlusion is preferable. Quantitative approaches however, applied to learn more about how specific design features of endovascular stents such as porosity [6], struts [7], and mesh design [8] affect intra-aneurysmal hemodynamic,s have mainly provided inconsistent results [9]. In some cases, stenting alone has been suggested to promote thrombogenic conditions such as reduced flow activity and prolonged stasi, and thereby occlude aneurysms simply by thrombosis.

But the selection of the preferred therapy is still controversially discussed. In this regard novel therapies such as flow dividers may also be considered [10]. For this reason blood flow simulations in the context of aneurysms of elastotypic and/or mixtotypic arteries have been proposed by various workgroups [11–13] and in different studies, for example, the ISAT study (International Subarachnoid Aneurysm Trial [14]). The Aneurist Project (http://www.aneurist.org/; as of april 1st, 2013), funded by the European Commission, is among the most renowned approaches. Their results [15, 16] state that a single simulation takes about 10 to 24 hours to complete. This does not involve testing different stent models, different placements, and varying orientations of the stent in the vessel. Such timing, however, is not helpful in a clinical setting. Computer-simulation-based therapy appears to be gaining acceptance in healthcare as several technical problems can be solved and facts be learnt without animal experimentation or by working with actual patients. The speed with which considerable quantities of simulations can be performed may reduce the number of animal experiments and identify new issues to be covered.

The present study has therefore been conducted to present a novel idea in combining the following different mathematical methods to quickly explore some of the above parameters: finite element techniques and lattice Boltzmann methods.

Finite element techniques represent the ubiquitous numerical method in structure and fluid mechanics. With its thorough theoretical background, error analysis for validation of simulation results can be achieved. Newer techniques such as lattice Boltzmann methods (LBM) provide no easy way to perform error analysis but may have advantages in different areas, for example, fast execution times. These fast execution times can be provided by using new programming paradigms for massively parallel processors such as graphics processing units (GPUs) available in most medical workstations. In order to explore giant parameter spaces, a combination of these methods may fuse the robustness of finite element results with the fast execution times of the other method.

LBM is a popular mesoscopic method in computational fluid dynamics. It has been applied to a number of interesting flow problems including multiphase and multicomponent fluid flows [17–19]. A relatively simple single-phase, single-component flow represents a good candidate for parameter exploring as it has been shown in the literature that the LBM approximates the time-dependent Navier-Stokes equations under certain circumstances [20]. The monographs [21, 22] are well-known starting points for further information; a GPU-specific discussion of LBM in the context of blood flow can be found in [23]. LBM models can be easily parallelized and therefore can be used to interactively explore different flow scenarios. The idea is that once an interesting set of boundary conditions and stent designs can be identified, highly accurate and highly detailed but much slower finite element simulations can be substituted and provide a more in-depth look.

The paper is organized as follows.  Section 2 introduces the simulation domains, the different numerical methods for simulation of blood flow and presents the concepts of finite element methods (Section 2.2) and lattice Boltzmann methods (Section 2.3). Following, Section 3 shows exemplary results that are obtainable using the presented methods for simulation and Section 4 concludes with some remarks on the current state and the further development.

## 2. Simulation of Blood Flow

 For evaluation and comparison purposes a set of basic conditions, that all simulation models have to comply with, is defined. These conditions have to be simple enough to allow the use of simplifying simulation models for faster access to initial simulation results, yet complex enough to model most aspects required for simulation of blood flow. Consequently, our finite element and lattice Boltzmann models consist of an incompressible or weakly compressible fluid modelling and a suitable viscosity model. In addition no slip boundary conditions and a maximum inflow velocity magnitude of 50 mm/s with a parabolic shape that is suitable for a small artery with a diameter of 3 mm are applied [24].

### 2.1. Datasets

**Figure 1:**
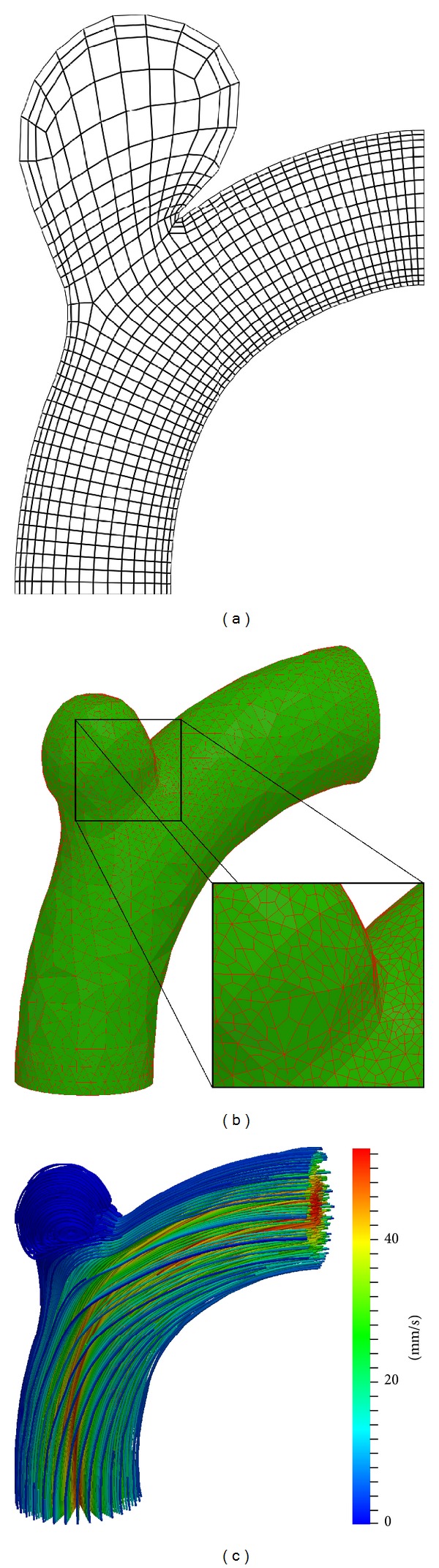
Simulation domain and border representation for (a) 2D FE mesh, (b) 3D FE mesh. (c) 3D stream line result for medium-sized aneurysm.

 For the purpose of comparing the different simulation models to each other an appropriate testing environment is needed. In addition to the meshes generated directly from MRI datasets, which sometimes suffer from irregularities and which are by concept limited to one stage in the formation process of an aneurysm, a synthetic model of a so-called true arterial aneurysm (syn.: Aneurysma verum), arbitrarily assumed to be similar to the terminal-type C morphology of unruptured aneurysms [25], was designed based on available MRI data and medical expert knowledge. Additionally, two hypothetical stages of aneurysm growth for the synthetic model are included in this study. The synthetic mesh facilitates the analysis of our physical modelling by providing well-structured 2D grids (cf.  Figure 1(a)), level set volumes (cf. [26, 27]) and 3D meshes (cf.  Figure 1(b)) for all required simulation domains.

### 2.2. Finite Element Method

 The solver used to perform the 2D calculations in this work is based on the ALE formulation of the Navier-Stokes equations; however to perform the 3D calculations it is modified in some important aspects. Instead of using an ALE formulation of the Navier-Stokes equations, an Eulerian approach is implemented. This approach is based on the incompressible Navier-Stokes equations, so the motion of an incompressible fluid at time *t* is governed by

(1) $$\begin{array}{c} \rho \left(\frac{\partial u}{\partial t}+u\cdot \nabla u\right)-\nabla \cdot \sigma =0,\;\nabla \cdot u=0\;\;\forall t\in \left(0,T\right), \end{array}$$

where *σ* is the stress tensor of the fluid phase:

(2) $$\begin{array}{c} \sigma =-pI+\mu \left[\nabla u+{\left(\nabla u\right)}^{T}\right]. \end{array}$$

We denote the identity tensor by *I*, the fluid density by *ρ*, the viscosity by *μ*, the pressure by *p*, and by **u** we refer to the fluid velocity. Space discretisation in 2D and 3D is then done by the FEM using the LBB stable conforming biquadratic, discontinuous linear *Q*_2_/*P*_1_ element pair. In time the equations are discretised using the Crank-Nicolson time-stepping scheme. The resulting system is then solved using a a standard geometric multigrid solver in 2D [28, 29] and a parallel Newton-multigrid solver in 3D [30].

### 2.3. Lattice Boltzmann Method

 In the last section, fluid behaviour is described by time-varying macroscopic fields. A microscopic point of view tracks the motion of each atom or molecule. The LBM takes a mesoscopic approach from statistical physics. Here, the (macroscopic) density *ρ* of a fluid is represented by multiple particle distribution functions (PDF) which represent fluid particles that move in the same direction. In the LBM, the directions are discretised onto a regular three-dimensional lattice. Each direction **e**_**i**_ linking a grid node with its neighbours corresponds to a PDF *f*_*i*_. The direction **e**_0_ is the zero vector which represents particles at rest. The discretisation in this case in three dimensions is commonly referred to as *D*3*Q*19 and consists of 19 directions, that is, *i* = 0,…, 18. In two dimensions a *D*2*Q*9 model with 9 discrete directions is used (details omitted, cf. [21]). The evolution of the PDFs at each lattice node with regard to collisions between fluid particles is described by (3) (see [22]). It holds

(3) fi(x+ei,t+1)−fi(x,t)  =−fi(x,t)−fieq(x,t)τ, i=0,1,…,18,

in which

(4) $$\begin{array}{c} f_{i}^{\text{eq}}=w_{i}\rho \left(1+3e_{i}\cdot u+\frac{9}{2}{\left(e_{i}\cdot u\right)}^{2}-\frac{3}{2}u\cdot u\right) \end{array}$$

are the 19 equilibrium distribution functions and *w*_*i*_ are weighting factors for the *DxQy* model. The evolution of the directional densities can be understood as a relaxation towards local equilibrium which is a function of the local density *ρ*, the current velocity **u**, and the relaxation time *τ* which is connected to the liquid viscosity *ν* = (1/3)(*τ* − 1/2). The equilibrium distribution functions *f*_*i*_^eq^ have the property to conserve mass as can be seen from (5). The density

(5) $$\begin{array}{c} \rho \left(x\right)=\sum_{i=0}^{18}f_{i}\left(x\right) \end{array}$$

at a lattice node is the sum of the PDFs in every direction. The current velocity

(6) $$\begin{array}{c} u\left(x\right)=\frac{1}{\rho }\sum_{i=0}^{18}f_{i}\left(x\right)\cdot e_{i} \end{array}$$

is also computed from the PDFs.

Solid boundaries can relatively easily be incorporated by swapping opposite PDFs at solid nodes. This technique known as bounce-back is one way of simulating the no-slip condition at solid boundaries. In the simulation of blood flow using LBM this bounce-back is used at the blood vessel boundaries and the stents. The structures themselves are defined by multiple-level sets [26]. A steady blood flow through the vessel is initiated by introducing pressure or velocity boundaries at the ends of the vessel. Here, velocity Dirichlet conditions at the inflow and velocity Neumann conditions at the outflow are applied; see [31] for further details. The compressibility error depends on the Mach number. With a Mach number *M* ≪ 1, the method is incompressible. It has been shown in the above literature that the lattice Boltzmann method approximates the time-dependent isothermal and incompressible Navier-Stokes equations under this circumstance. So in theory, the above finite element ansatz and the LBM should yield comparable results.

## 3. Results

 Based on available real geometry data of blood vessels featuring an aneurysm and our synthetic aneurysm models, some basic simulations are performed to compare the simulation methods. For FEM, the 2D quad meshes consist of 4,208–4,244 elements with *≈*81,000 degrees of freedom and the level 1/2 3D hexahedral mesh consists of 26,177/173,600 elements with *≈*2.1/14 Mio unknowns. Lattice sizes for LBM are 272 × 384 in 2D and 188 × 88 × 212 in 3D, respectively, that is, *≈*3.44 Mio active *D*3*Q*19 cells with *≈*65.2 Mio PDFs. The simulations are parameterized for a channel width of 3 mm, a parabolic velocity profile with a maximum velocity of 50 mm/s, a density of 1060 kg/m^3^, and a dynamic viscosity of 0.004 kg/ms. The resulting Reynolds number is 19.88.

**Figure 2:**
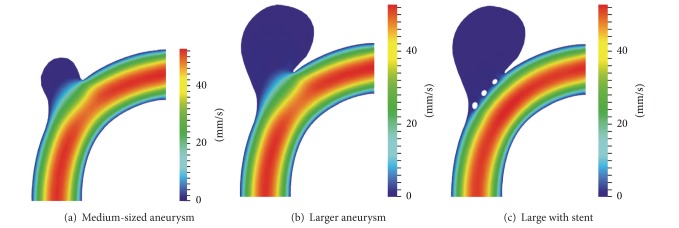
Visualization of the 2D FEM velocity field in an aneurysm bearing artery: (a), (b) nonstented case and (c) with coarse stent.

**Figure 3:**
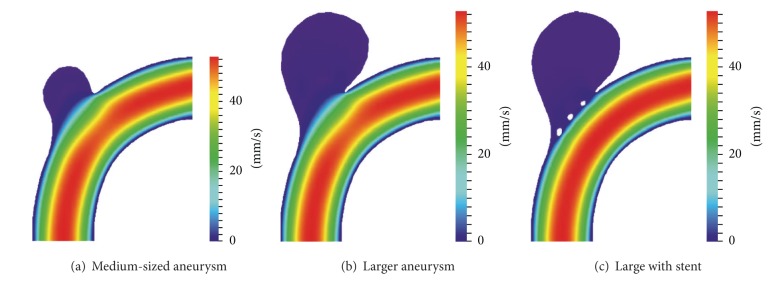
Visualization of 2D LBM velocity field in an aneurysm bearing artery: (a), (b) nonstented case and (c) with coarse stent.

**Figure 4:**
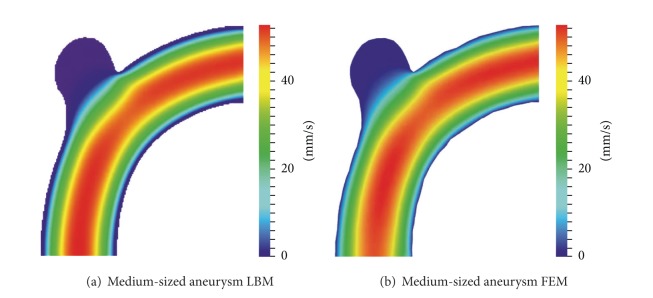
Visualization of a 2D cut through the middle of the 3D velocity field in an aneurysm-bearing artery: (a) LBM, (b) FEM.

**Figure 5:**
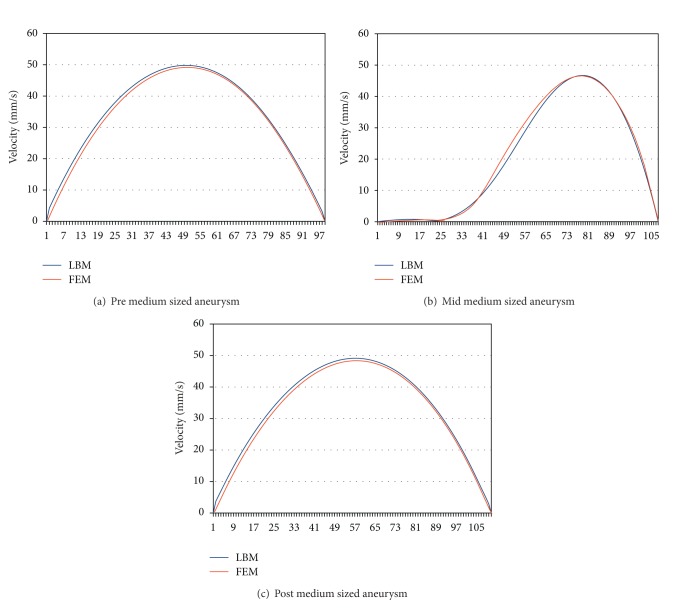
Medium-sized aneursym: stationary stream profiles of the 2D simulations (a) in front of, (b) at and (c) after the aneurysm neck. The numbered sampling points are displayed on the *x*-axis. For (a), (c) the length of the cutline is 3 mm, for (b) 5 mm.

**Figure 6:**
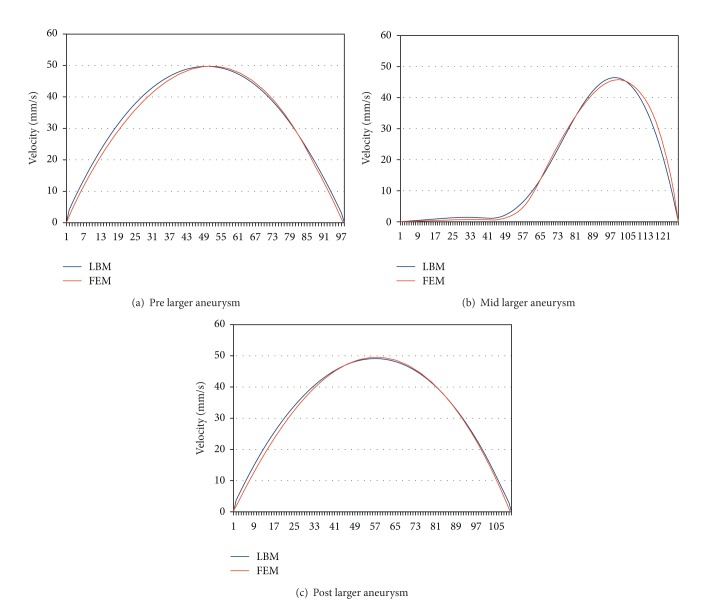
Larger aneurysm: stationary stream profiles of the 2D simulations (a) in front of, (b) at, and (c) after the aneurysm neck. The numbered sampling points are displayed on the *x*-axis. For (a), (c) the length of the cutline is 3 mm, for (b) 6 mm.

**Figure 7:**
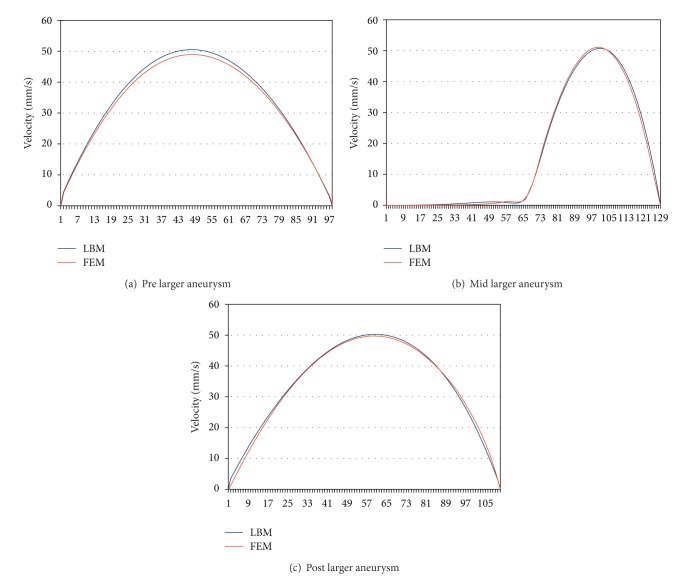
Stented larger-sized aneurysm: stationary stream profiles of the 2D simulations (a) in front of, (b) at, and (c) after the aneurysm neck. The numbered sampling points are displayed on the *x*-axis. For (a), (c) the length of the cutline is 3 mm, for (b) 6 mm.

**Figure 8:**
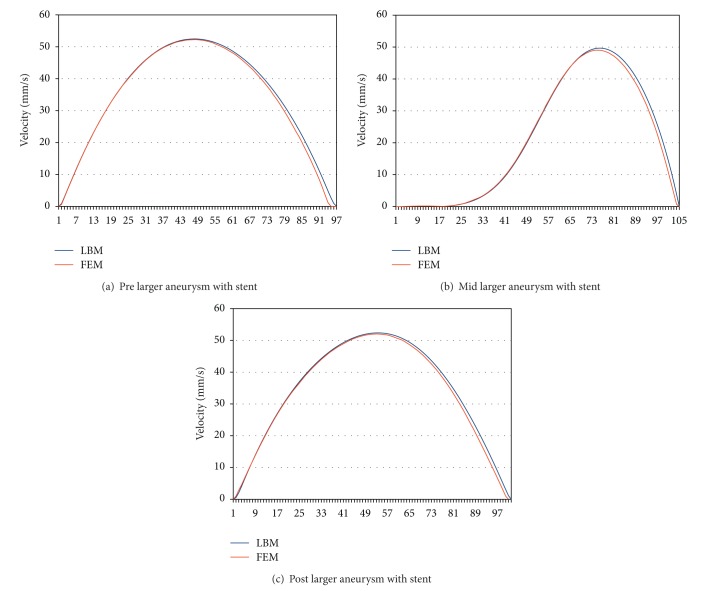
Medium-sized aneurysm: stationary stream profiles of the 3D simulations for the same plane as 2D data set: (a) in front of, (b) at, and (c) after the aneurysm neck. The numbered sampling points are displayed on the *x*-axis. For (a), (c) the length of the cutline is 3 mm, for (b) 5 mm.

To analyse aneurysm growth and its influence on the flow fields, we perform some basic tests using the two stages of our synthetic aneurysm model from Figure 1. In Figure 1(c) a streamline view of the 3D case is shown. The velocity fields obtained with the FEM and LBM models are shown color-coded in Figures 2, 3, and 4. Comparisons of three cutlines in 2D and the midline of three cut-planes in 3D (same location as in 2D) can be found in Figure 5 (2D FEM and LBM medium-sized aneurysm), Figure 6 (2D FEM and LBM larger aneurysm), Figure 7 (2D FEM and LBM large with stent), and Figure 8 (3D FEM and LBM medium aneurysm). The cutlines/-planes are located in the vessel before the aneurysm neck (“pre”), at the aneurysm neck at a 45 degree angle to the curvature of the vessel (“mid”), and after the aneurysm neck (“post”). The results of all unstented simulation models share a (deformed) parabolic velocity profile throughout the blood vessel, a drop in velocity magnitude near the opening of the aneurysm, a widening of the parabolic profile, and a significant velocity magnitude at the aneurysm neck. The larger the aneurysm the higher the drop in magnitude in the vessel at the neck. Comparing the results inside the vessel with those inside the aneurysm, no such high velocity magnitudes do occur. On average the velocity magnitude is only *≈*1 mm/s whereas at the aneurysm neck the velocity is *≈*14–20 mm/s depending on the model used. The differences in velocity magnitude of the different numerical methods are low.

A comparison of the stented vessel with its nonstented counterpart can be found in Figures 2(c), 3(c), 6, and 7. It can be seen that much of the inflow at the aneurysm neck is effectively disabled by the stent. The average velocity inside the aneurysm drops from *≈*1 mm/s in the nonstented case to *≈*0.75 mm/s. The flow behaviour of all simulations is nearly identical. The fluid streams from the vessel into the aneurysm lumen through the first three stent gaps and leaves the aneurysm sack through the fourth gap. The velocity magnitude drops from *≈*14 mm/s in the nonstented case to *≈*5 mm/s in the stented case at the neck.

Regarding the initial goal of fast exploration of the parameter space running times are listed here. For 2D LBM and the shown data sets, we recorded approximately 1900 LBM iterations per second on a NVIDIA 560Ti GTX and approximately 2600 iterations per second on a NVIDIA 680GTX graphics card while 1 s equals 16667 LBM time steps. In 3D and with 3.44 Mio cells, we record 71.8 and 118.3 iterations per second with the two graphics cards. With simultaneous volume visualization of the velocity field, these numbers drop to 58.2 and 88.3 iterations per second. A simulation of a cardiac cycle with a duration of 1 s equals 11667 3D LBM iterations in this parametrization. It can be simulated in under 2 min. Compared to 11 hours for 1 s with 2500 time steps on 32 processors for the 3D FEM, exploration of multiple scenarios seems possible. Note that using adaptive time step sizes for FEM can reduce the execution times to 50% or less of the aforementioned value for the test case under consideration.

## 4. Discussion

 The presented results show that the mathematical construction of patient-specific anatomy is both feasible and applicable to realistic test cases. Various practical issues have to be considered in order to establish a tailor-made aneurysm therapy based on mathematical modelling to implement personalized stenting for individual patients based on clinical and radiological findings.

In this comparative analysis of different methods, the FEM approach is the most expressive model at the moment. Because of its high complexity, the computation time is comparably slow and is usually a matter of hours or even days. But it is possible to resolve the fine-scale features of the flow by increasing mesh resolution or by local mesh adaptation. It seems reasonable to use an additional simulation method with very comparable results but with specific advantages for interactive parameter exploring. A comparison of both methods using the configuration described in Section 3 is provided in Table 1. Interesting flow constellations can be further analysed by the FEM after this initial exploration.

Due to the inherent parallelism of the LBM, where computation in each lattice node is only dependent on a local neighbourhood, the algorithm can be performed on highly parallel computing architectures such as graphics processors. This approach has been taken in the reference implementation which uses OpenCL (Open Computing Language) for computation. In the test case described in this work a parallel FEM implementation on 32 cores is outperformed by a factor of 100–400 with recent NVIDIA Kepler GPU architectures and very well-comparable results. The interactive frame rates of parallel LBM simulations can provide key simulation constellations that can be investigated further with complex time-dependent nonNewtonian fluid structure interaction models. The influence of far-reaching and concentrated inflow jets on the integrity of the aneurysm sack has not been conclusively determined although some results and investigations exist [16].

On the basis of this introductory study it can be concluded that the time-dependent flow characteristics have to be analysed as well as the stationary results. Besides the above mentioned technical aspects, an optimal flow diverting stent geometry has to be found for the cardiac cycle because in the stationary case even the most basic stent is able to do its job after some time steps. Comparing the results of the rather simple stent model provides no clear tendency for the influences of the inflow jets other than lower average velocities inside the aneurysm.

But these results indicate already that a multidisciplinary approach to the development of individualized aneurysm therapy is feasible and should be applied in the early development stages of novel stenting devices. Both of the approaches evaluated in the present publication encourage increasing use of numerical simulation in the development process of novel stenting devices. Especially considering that future mathematical models may allow for more features of the blood flow to be evaluated (e.g., thrombosis), such models are a part of future research activities.

In the stented case, substantial indications have been given for areas of zero velocity and without rotational behaviour in the periphery. An additional thrombosis model could be implemented to analyse thrombus growth in these regions.

The developed methods have to be refined in such a way that they provide the necessary resolution and respective pulsatory behaviour, so they are able to interact with boundary geometry and are able to model growth as well as modify other relevant parameters such as thrombosis parameters in order to automatically determine a stent geometry that is best for a specific situation. Tools for 2D/3D blood flow visualization are not only useful to showcase results of numerical computations but also to offer great help to doctors and medical professionals in the treatment of the corresponding health problems as these tools provide new information that is not accessible using traditional tools. The future research activities of this research group focus on the analysis and development of patient-specific stent geometries or to alternatively provide a software-assisted stent geometry recommendation from a set of clinically available stents.

## Figures and Tables

**Table 1:** Comparison of different simulation methods.

Aspect	FEM	LBM
Ansatz	Euler	Euler
Incompressibility	Yes	Yes
Time steps	Large	Implicit scheme: medium
Cost per time step	Large	Small
Boundary-fitted domain	Difficult	Fine grids
Level-set-based domain	Research topic, fictitious domain techniques	Research topic, same grid
Error analysis	Yes	Partial
Error control	Yes	No
Mesh/grid refinement	Yes	Research topic
Accuracy	High	Good
Non-Newtonian rheology	Yes	Available
Thrombosis model	Research topic	Research topic
Fluid structure interaction	Research topic	Research topic
Turbulence	Partial	Partial
Code implementation	Complicated	Good
Memory usage	Q2P1: high	D3Q19: high
Parallelisation	Complicated	Good

## References

[1] Joo SW, Lee SI, Noh SJ, Jeong YG, Kim MS, Jeong YT (2009). What is the significance of a large number of ruptured aneurysms smaller than 7 mm in diameter?. *Journal of Korean Neurosurgical Society*.

[2] Lu J, Liu JC, Wang LJ, Qi P, Wang DM (2012). Tiny intracranial aneurysms: endovascular treatment by coil embolisation or sole stent deployment. *European Journal of Radiology*.

[3] Rinkel GJE (2008). Natural history, epidemiology and screening of unruptured intracranial aneurysms. *Journal of Neuroradiology*.

[4] Chien A, Sayre J, Vintildeuela F (2011). Comparative morphological analysis of the geometry of ruptured and unruptured aneurysms. *Neurosurgery*.

[5] Morita A, Kimura T, Shojima M, Sameshima T, Nishihara T (2010). Unruptured intracranial aneurysms: current perspectives on the origin and natural course, and quest for standards in the management strategy. *Neurologia Medico-Chirurgica*.

[6] Aenis M, Stancampiano AP, Wakhloo AK, Lieber BB (1997). Modeling of flow in a straight stented and nonstented side wall aneurysm model. *Journal of Biomechanical Engineering*.

[7] Lieber BB, Livescu V, Hopkins LN, Wakhloo AK (2002). Particle image velocimetry assessment of stent design influence on intra-aneurysmal flow. *Annals of Biomedical Engineering*.

[8] Liou TM, Liou SN, Chu KL (2004). Intra-aneurysmal flow with helix and mesh stent placement across side-wall aneurysm pore of a straight parent vessel. *Journal of Biomechanical Engineering*.

[9] Kim M, Taulbee DB, Tremmel M, Meng H (2008). Comparison of two stents in modifying cerebral aneurysm hemodynamics. *Annals of Biomedical Engineering*.

[10] Kamran M, Yarnold J, Grunwald IQ, Byrne JV (2011). Assessment of angiographic outcomes after flow diversion treatment of intracranial aneurysms: a new grading schema. *Neuroradiology*.

[11] Gambaruto AM, Janela J, Moura A, Sequeira A (2011). Sensitivity of hemodynamics in a patient specific cerebral aneurysm to vascular geometry and blood rheology. *Mathematical Biosciences and Engineering*.

[12] Yoshimoto Y (2006). A mathematical model of the natural history of intracranial aneurysms: quantification of the benefit of prophylactic treatment. *Journal of Neurosurgery*.

[13] Chang HS (2006). Simulation of the natural history of cerebral aneurysms based on data from the International Study of Unruptured Intracranial Aneurysms. *Journal of Neurosurgery*.

[14] Molyneux A, Kerr R, Stratton I (2002). International Subarachnoid Aneurysm Trial (ISAT) of neurosurgical clipping versus endovascular coiling in 2143 patients with ruptured intracranial aneurysms: a randomised trial. *Lancet*.

[15] Appanaboynia S, Mut F, Löhner R, Putman C, Cebral J (2009). Simulation
of intracranial aneurysm stenting: techniques and challenges. *Computer Methods in Applied Mechanics and Engineering*.

[16] Cebral JR, Mut F, Weir J, Putman CM (2011). Association of hemodynamic characteristics and cerebral aneurysm rupture. *American Journal of Neuroradiology*.

[17] Inamuro T, Ogata T, Tajima S, Konishi N (2004). A lattice Boltzmann method for incompressible two-phase flows with large density differences. *Journal of Computational Physics*.

[18] Yuan P, Schaefer L (2006). Equations of state in a lattice Boltzmann model. *Physics of Fluids*.

[19] Shan X, Chen H (1993). Lattice Boltzmann model for simulating flows with multiple phases and components. *Physical Review E*.

[20] Junk M, Yong WA (2003). Rigorous Navier-Stokes limit of the lattice Boltzmann equation. *Asymptotic Analysis*.

[21] Succi S (2001). *The Lattice Boltzmann Equation for Fluid Dynamics and Beyond*.

[22] Sukop MC, Thorne DT (2006). *Lattice Boltzmann Modeling: An Introduction for Geoscientists and Engineers*.

[23] Walczak L, Fisseler D, Weichert F, Goltz U, Magnor MA, Appelrath H-J, Matthies HK, Balke W-T, Wolf LC (2012). Exploring therapy options with the interactive simulation of intra-aneurysmal blood flow on the gpu. *GI-Jahrestagung*.

[24] Speckmann E-W, Hescheler J (2008). *Physiologie*.

[25] Ohshima T, Miyachi S, Hattori KI (2008). Risk of aneurysmal rupture: the importance of neck orifice positioning-assessment using computational flow simulation. *Neurosurgery*.

[26] Sethian JA (1999). *Level Set Methods and Dynamic Implicit Surfaces—Evolving Interfaces in Computational Geometry, Fluid Mechanics, Computer Vision, and Materials Science*.

[27] Walczak L, Weichert F, Schröder A, Landes C, Müller H, Wagner M (2009). Evaluating the impact of shape on finite element simulations in a medical context. *Modelling the Physiological Human*.

[28] Razzaq M, Damanik H, Hron J, Ouazzi A, Turek S (2011). FEM multigrid techniques for fluid-structure interaction with application to hemodynamics. *Applied Numerical Mathematics*.

[29] Razzaq M (2011). *Finite element simulation techniques for incompressible fluid-structure interaction with applications to bio-engineering and optimization [Ph.D. thesis]*.

[30] Münster R, Mierka O, Turek S (2012). Finite element-fictitious boundary methods (FEM-FBM) for 3D particulate flow. *International Journal for Numerical Methods in Fluids*.

[31] Zou Q, He X (1995). On pressure and velocity flow boundary conditions for the lattice boltzmann bgk model. *Biophysics*.

